# A Conservative Management of Perforated Peptic Ulcer: A Case Report

**DOI:** 10.7759/cureus.56491

**Published:** 2024-03-19

**Authors:** Haitham H Abdalgalil, Ahmed S Ismail, Hanan O Alshmaily, Dalal S Alshammari

**Affiliations:** 1 General Surgery, King Khalid Hospital, Hail, SAU; 2 Medicine, University of Hail College of Medicine, Hail, SAU

**Keywords:** nsaids, gas under diaphragm, conservative management, perforated hollow viscus, perforated peptic ulcer

## Abstract

Peptic ulcer disease (PUD) is a surgical emergency that affects the mucosal lining of the stomach or proximal intestine. Complications of PUD include upper gastrointestinal hemorrhage, perforation, and obstruction. The primary management approach for perforated peptic ulcers is surgery, but conservative management can be conducted in selected cases.

A 54-year-old female was referred to the surgical unit with a history of severe upper abdominal pain and repeated vomiting. No other symptoms were reported and there was no significant medical or family history except the history of non-steroidal anti-inflammatory drugs.

Examination revealed that the patient had a medical condition. was vitally stable with tenderness in the upper abdomen, in particular the epigastric and right hypochondrial, but no signs of generalized peritonitis. Her white cell count was elevated at 24,000x10^3/UL, and a C-reactive protein of 45.5 mg/dL. An upright CXR revealed the classic gas under the diaphragm. Abdominal CT with oral gastrograffin identified the diagnosis of perforated duodenal ulcer without ulcer leak. The case was treated by conservative management started with resuscitation, nil per os, IV fluid, IV antibiotics, and close observation and the patient was stable with no complications and completed the nonoperative management successfully till discharge after 10 days of hospital stay. The case illustrates that although this condition is uncommon to be treated without surgical intervention, there are some factors and criteria for successful NOM.

Peptic ulcer perforation is a life-threatening surgical emergency. Surgery is the standard treatment for PPU and NOM can be conducted safely and successfully in highly selected cases. the surgeon should keep a wide safety window while providing nonstandard management with readiness to operate at any time. We believe that the main factor in successful nonsurgical management of our case is being fasted for a long time before perforation.

## Introduction

Peptic ulcer disease (PUD) occurs when the mucosal lining of the stomach or proximal intestine is affected. Individuals who engage in smoking, use non-steroidal anti-inflammatory medicines (NSAIDs), have an infection with Helicobacter pylori, or lead a sedentary lifestyle are at an increased risk of developing PUD. The age of the patient and the location of the ulcer-gastric or duodenal affect the clinical manifestations of the disease. However, in general, patients who present with burning, postprandial fullness, or early satiety raise the suspicion of PUD [1,2]. Upper gastrointestinal bleeding, perforation, and blockage are among PUD complications. When a peptic ulcer is perforated, gastric juice and gas enter the peritoneal cavity, causing peritonitis. The patient typically presents with abrupt onset of abdominal discomfort, tachycardia, and rigidity. Surgery is the main method of treating a perforated peptic ulcer, while conservative measures may also be used in some circumstances [3,4]. We present a cautious approach to treating a patient with a ruptured duodenal peptic ulcer in this article.

## Case presentation

A 54-year-old Saudi woman who had just gone through menopause arrived early in the morning at the King Khalid Hospital's Emergency Department in Hail City. She had been experiencing moderately severe upper abdominal pain and vague pain for the previous two weeks, along with frequent vomiting for three days. About 12 hours before arriving at the emergency room, the pain worsened and became more confined to the right upper abdomen and epigastrium, accompanied by frequent vomiting. The patient reported a lengthy history of using NSAIDs often to treat arthralgia. She has never had surgery before and is currently on antihypertensive drugs.

During the examination, the patient was experiencing intense pain and remained fully conscious. Their vital signs were as follows: heart rate of 96 beats per minute, blood pressure of 110/70, respiratory rate of 24 breaths per minute, and oxygen saturation level of 94%. The patient's vital signs were stable, and they did not have a fever. The abdomen felt soft and loose, with notable discomfort in the epigastric and right hypochondrial regions.

Immediate resuscitation in the Emergency department involved the use of omeprazole, IV hydration, and IV analgesics. An examination in the lab showed signs of Acute Kidney Injury and increased WBC (Table 1). ABG showed compensatory acidosis, a base deficit of 3.4, and a lactate of 1.6 in the serum.

**Table 1:** Lab test

Test Name	Results	Normal Range	Units
White cell count	24.34	3.5-10	10^3/UL
C-reactive protein	45.5	0.3-1.0	mg/dL
Serum lactate	1.8	<1.0	mmol/L
Creatinine	227	53-97.2	Umol/L
Urea	12.8	2.7 – 8.5	mmol/L
Serum calcium	2.43	2.12 - 2.52	mmol/L

**Figure 1:**
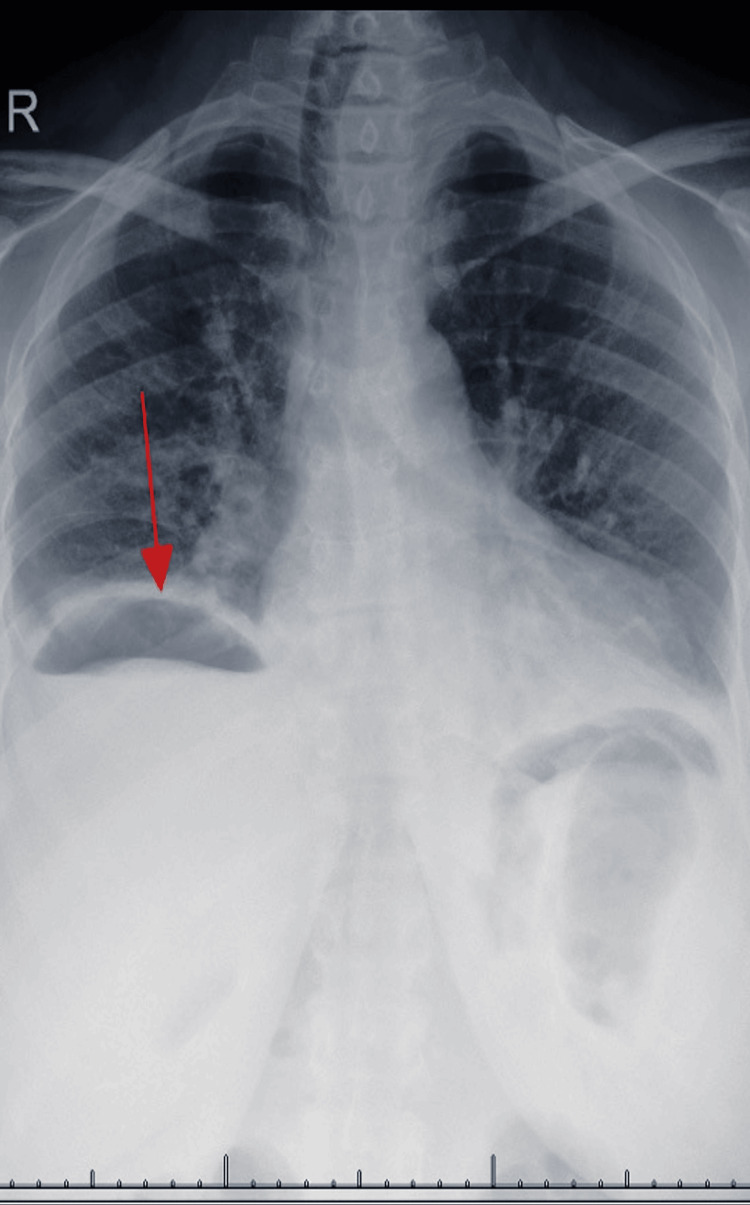
Chest x-ray upright showed evidence of pneumoperitoneum

Was an upper gastrointestinal endoscopy performed to identify the perforated site? The first choice to perform a laparotomy was based on the x-ray diagnosis of considerable pneumoperitoneum (Figure 1), which was conventionally straightforward. The patient was counseled and given consent.

**Figure 2:**
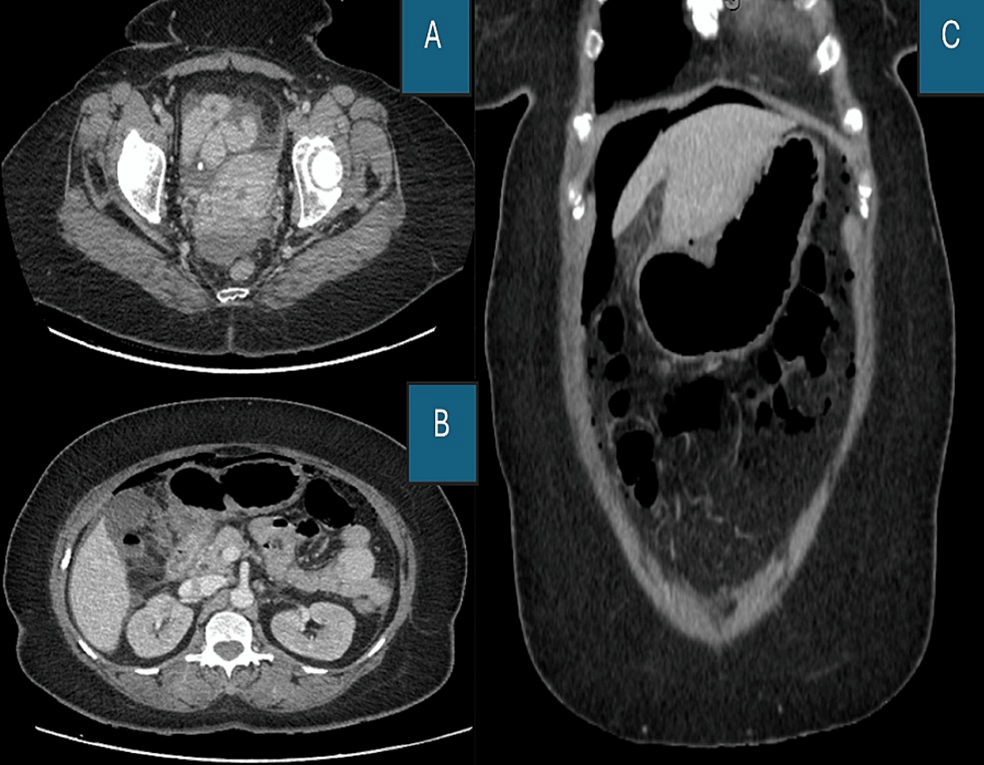
Abdominal CT with oral gastrograffin (A) CT axial cut showing mild pelvic fluids, (B) CECT axial cut of upper abdomen showing periduodenal mild fluids and oedema without contrast leak, (C) Abdominal CT coronal cut showing subdiaphragmatic perihepatic free air

With numerous calls for road traffic accidents, damage control laparotomy, and three significant acute abdominal cases during the sweltering night, there was no time to consider surgical alternatives. Despite knowing that the patient was obese, the treating consultant and his team reevaluated the patient after the morning meeting while they waited in the operating room. They discovered that the patient was comfortable, had little pain, was vitally stable, had a pulse rate of about 92, normal blood pressure, good urine output, and showed no signs of generalized peritonitis. The prospect of considering conservative therapy following the CT-based exclusion of active leakage with oral gastropraffin persuaded the entire team. The CRP was 43 and the follow-up ABG was normal. No free leak was detected by CT using oral contrast (Figures 2A-2C).

Because the patient was stable and improving following first resuscitation, had no extraluminal contrast leak in the CT scan, no widespread peritonitis, and a negligible CRP level, the procedure was canceled, and the patient was given advice regarding the revised treatment plan.

Her treatment plan included stringent nil per os (NPO), 3 L O2 with nasal cannula, IV fluids, omeprazole IV, broad-spectrum anti-microbial agents, close observation, many bedside examinations (General, abdominal examination), multiple labs including Full blood count, WBC, RBC, ABG, C-reactive protein, Serum lactate, Creatinine, Urea, Serum calcium, and is ready to operate at any time.

The patient had a smooth and improving course, with all parameters returning to normal within 24 to 48 hours after admission, with the exception of the WBC and creatinine levels. After 48 hours, we were not concerned about developing peritonitis; instead, we were considering the likelihood of a local abscess forming from potential food particles inside the peritoneal cavity. In the event that we became suspicious of such a situation, we had a plan to perform laparoscopic peritoneal lavage. This led us to ask the patient about her last meal prior to the anticipated perforation incident. Fortunately, we discovered that she had been close to fasting for several days due to pain and vomiting.

**Figure 3:**
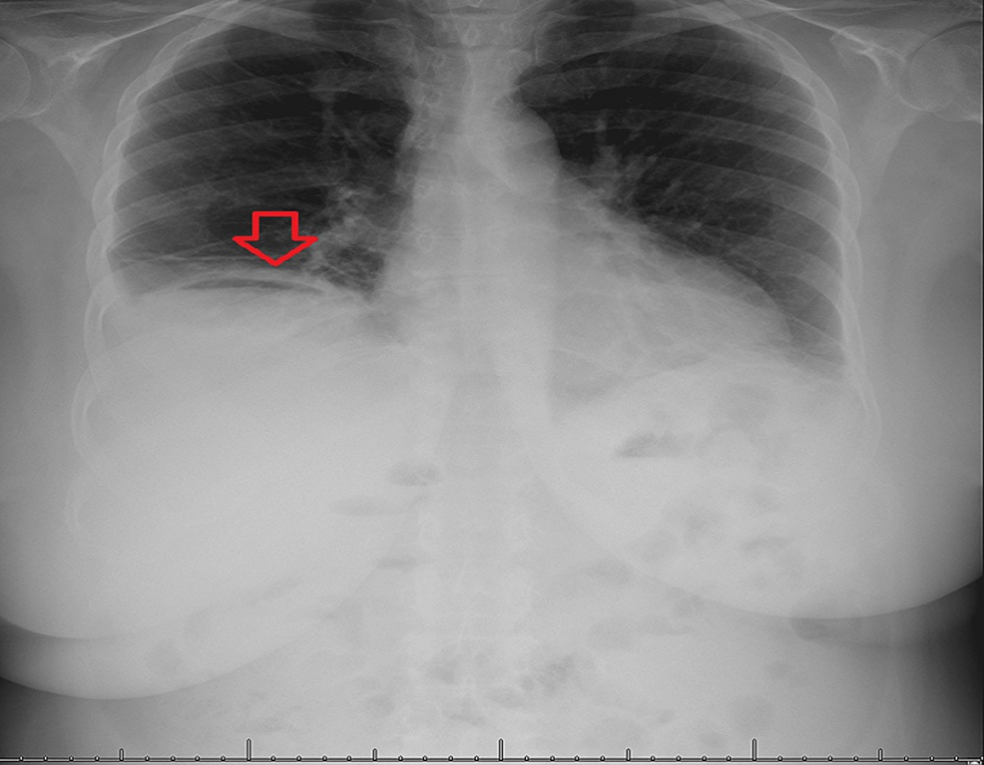
Chest x-ray before discharge revealed minimal residual air

On the fourth day, the patient was admitted and provided access to clear oral fluids for a duration of two days. After that, they were allowed to continue the regular diet, and on day 10, they were released from the hospital taking omeprazole with a scheduled outpatient follow-up appointment for endoscopic and general surgery. Prior to release, an x-ray and US were conducted, and the results showed very little remaining fluid and air (Figure 3).

## Discussion

PUD is still common in the general population, with a prevalence of around 5%-10% and a rate of 0.1%-0.3% per year, even though there have been recent advances in both diagnostic and therapeutic approaches. Patients who tend to be elderly with multiple co-morbidities and associated use of non-steroidal anti-inflammatory drugs (NSAIDs) are higher risk factors for PPU [5,6]. Our patient had HTN and chronic use of NSAIDs.

Perforation of peptic ulcer remains the primary cause of gastrointestinal perforation and one of the most frequent complications of PUD [6]. It is a life-threatening surgical emergency with a mortality risk ranging from 1.3% to 20% [7,8]. The initial symptoms of a perforated gastric or duodenal ulcer may be atypical, and clinical observation may not be possible in the case of comorbidities [8-10].

The clinical presentation of these perforations in elderly patients is often different from the classical picture. The sudden onset of severe pain with board-like abdominal rigidity frequently does not occur and it tends to be more insidious [8,11].

A radiograph of the abdomen and chest is used during the initial diagnostic investigation to confirm the presence of free abdominal air. X-ray has a high diagnostic accuracy in the positions of erect and left lateral decubitus. The presence of this radiological sign is highly variable across various studies present in literature and ranges between 30% and 85% of perforations.

Our patient had minimal abdominal air in x-rays which in the context of the clinical scenario supported the diagnosis of PPU. CT scan findings in PPU included unexplained intraperitoneal fluid, pneumoperitoneum, bowel wall thickening, mesenteric fat streaking, and presence of extraluminal water-soluble contrast; however, up to 10% of patients with perforations may have a normal CT scan [12-14].

In the case of our report, we performed CT with oral contrast and showed there is a significant amount of the pneumoperitoneum with mild free fluid, likely related to viscous perforation. The radiologist reported that there was edematous thickening of the first part of the duodenum with multiple free air pockets seen adjacent to the first part of the duodenum and in the lesser sac, raising the possibility of a source of pneumoperitoneum to be a perforated duodenal wall. No contrast leak outside the GIT lumen could be seen.

Patients with clinical presentation of perforated peptic ulcer should be resuscitated even before the diagnosis is made. This is conducted through crystalloids, early intravenous antibiotics (especially if there are signs of SIRS), and analgesics. When the diagnosis of PPU is confirmed, the patient should be given proton pump inhibitors, IV antibiotics, and inserted nasogastric tube, with a surgical assessment, then the decision can be made regarding whether the patient will require surgery [15].

The standard management of PPU is surgery. A type 2 C recommendation from WSES guidelines is recommended against non-operative management (NOM) except in extremely selective cases. NOM can be conducted in a vitally stable patient with evidence of a sealed ulcer by water-soluble contrast study, and when there is no clinical evidence of peritonitis or sepsis [16].

In our case, the patient was vitally stable after fluid resuscitation with a pulse of 92, BP of 110/70, RR of 22, and oxygen saturation of 94%. CT with oral contrast revealed no free leakage, and there were no signs of peritonitis. Surgical management of patients with PPU is strongly recommended in patients with serious pneumoperitoneum or extraluminal contrast leak or clinical evidence of peritonitis. In patients over 70 years old or with delayed presentation, the surgery is recommended to be performed as soon as possible. Laparoscopy is suggested, safe, and feasible with experienced hands with superior outcomes regarding post-operative pain, wound complications, and hospital stay [16]. In our case, the patient was matching the criteria for NOM, so it was conducted safely and successfully.

## Conclusions

Perforation of a peptic ulcer is a surgical emergency that still carries a risk of mortality. Surgery is the standard treatment for PPU and NOM can be conducted safely and successfully in highly selected cases. the surgeon should keep a wide safety window while providing non-standard management with readiness to operate at any time. Surgical management of patients with PPU is strongly recommended in patients with significant pneumoperitoneum.
